# A Confused Case Diagnosed as Cerebral Infarction or Myelin Oligodendrocyte Glycoprotein Antibody-Associated Disease

**DOI:** 10.1155/2024/9941341

**Published:** 2024-08-23

**Authors:** Yanan Ding, Li Zhang, Anqi Huang, Xianyue Meng, Xueli Li

**Affiliations:** Department of Neurology Liaocheng Hospital Affiliated to Shandong First Medical University (Liaocheng People's Hospital), Liaocheng 252000, Shandong, China

## Abstract

In order to discuss the clinical and MRI features, diagnosis, and prevention of myelin oligodendrocyte glycoprotein antibody-associated disease (MOGAD), we reported an adult case of MOG antibody-related disease misdiagnosed as cerebral infarction. This patient's first clinical symptom was limb weakness, that different from previous reports of MOG antibody-related diseases, such as brainstem encephalitis, neuromyelitis optical, and transverse myelitis. The main treatment plan is high-dose corticosteroid therapy combined with immunoglobulin therapy. This case indicated that some MOGAD patients lack of specificity in the clinical manifestations and imaging perhaps would be misdiagnosed as cerebral infarction, encephalitis, immune peripheral neuropathy, MS, NMOSD, and other diseases. For patients with atypical clinical manifestations or imaging, it is especially important to take antibody detection as early as possible to make correct diagnosis and active treatment in time to avoid disability.

## 1. Introduction

Myelin oligodendrocyte glycoprotein (MOG) antibody-associated disease (MOGAD) is a recently characterized inflammatory demyelinating disorder that is mediated by autoimmune antibodies. Scientific research on MOG protein can be traced back to 1980, when it was first identified as the main potential autoantibody target of the CNS myelin in an experimental autoimmune encephalomyelitis model [1]. In 2003, western blotting was used to detect MOG-IgM antibody, that could be used as a biomarker to predict the transformation of Clinically Isolated Syndrome to definite MS [2]. However, in 2007, O'Connor found MOG-IgG antibody-related subgroups in patients with acute disseminated encephalomyelitis (ADEM) and optic neuritis (ON), but did not find it in MS patients [3]. Subsequent research by Ketelslegers confirmed the presence of MOG-IgG antibodies in non-MS demyelinating CNS disease [4]. MOGAD is a distinct disease spectrum different from MS and NMOSD [5, 6]. The main symptoms include ON, meningoencephalitis, brainstem encephalitis, and myelitis [7]. Few cases presented as cerebral infarction. We reported the patient with MOGAD that was initially diagnosed as cerebral infarction.

## 2. Case Description

**Figure 1:**
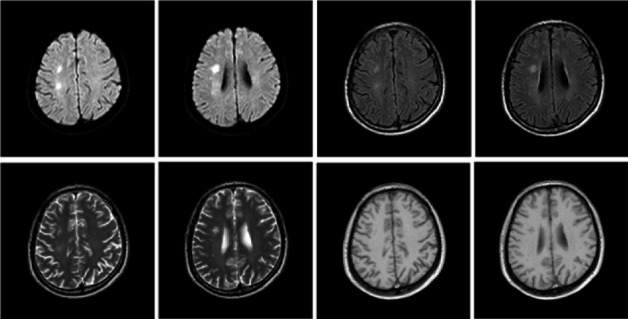
Head MRI (January 16, 2018 in Liaocheng People's Hospital) showed hyperintense lesions on DWI or hyperintense lesions on T2WI in the right semiovale center and radiating corona.

**Figure 2:**
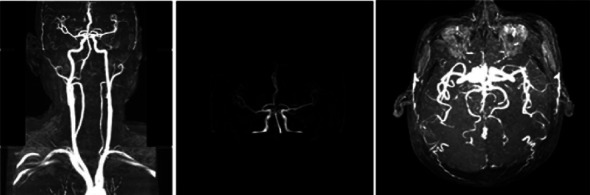
MRA of the head and neck (January 16, 2018): the left vertebral artery is slender throughout, and the blood flow signal at the end segment is weak, which is considered to be stenosis; the P3-4 segment of the posterior cerebral artery is limitedly stenotic.

**Figure 3:**
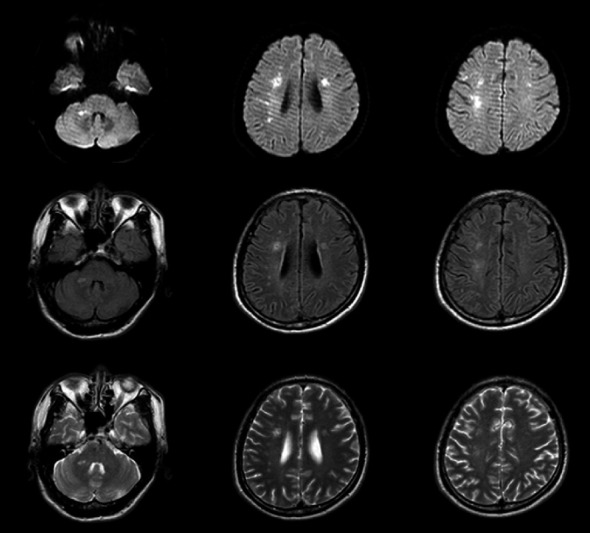
Head MRI (January 25, 2018 in Liaocheng People's Hospital) showed multiple new focus in bilateral semiovale center, corona radiata, and right bridge arm.

**Figure 4:**
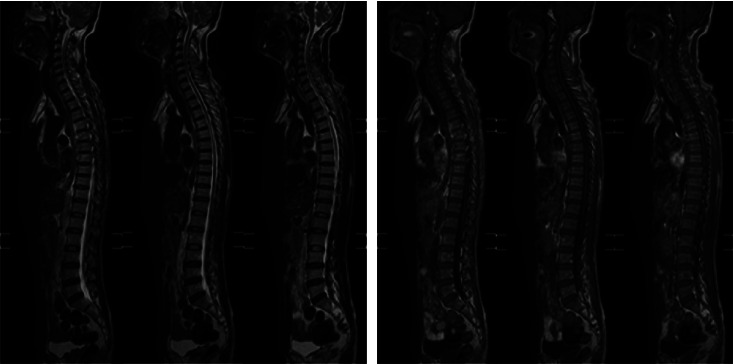
Magnetic resonance imaging of the whole spine (January 28, 2018): C4/5-C6/7 discs protrude backward, ligamentum flavum is hypertrophic, and the spinal canal is slightly narrowed; L4/5, L5/S1 discs protrude backward; degenerative changes of the entire spine.

**Figure 5:**
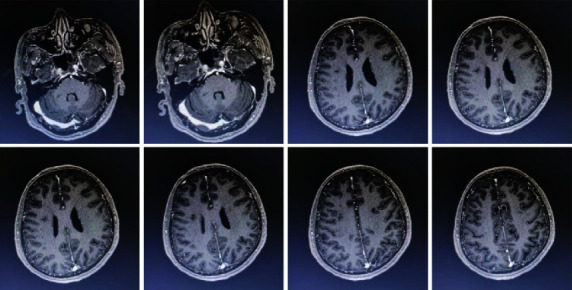
Cerebral MR enhancement (February 9, 2018): multiple abnormal signals in the brain without obvious enhancement.

The patient was a 60-year-old workman without disease before and without family disease history. He was admitted to hospital on January 16, 2018 because of a 1-month history of dizziness that had aggravated. It manifested as unsteady walking accompanied by nausea without vomit. Examination showed a clear consciousness, influent speech, and left central facial tongue paralysis; his left limb muscle strength was level 4. There were no obvious abnormalities in blood biochemistry, coagulation mechanism, virus screening, urine routine, glycosylated hemoglobin, fecal occult blood, and cardiac ultrasound but mild anemia. Head magnetic resonance imaging (MRI) showed hyperintense lesions on DWI or hyperintense lesions on T2WI in the right oval center and radiating corona (Figure 1). An uniformly thin left vertebral artery and weak blood flow signal in the terminal segment. The P3-4 segment of the posterior cerebral arterystenosiswas stenotic (Figure 2). The patient was diagnosed as cerebral infarction and treated with drugs such as antiplatelet therapy (Aspirin, promoting blood circulation and silting), Atorvastatin to stabilize plaque, and other drugs to improve symptoms. But nine days later, his symptoms worsened, he showed memory loss, poor speech, and apraxia. Both limb muscle strength level was level 4. A new head MRI showed multiple new focus on bilateral centrum oval, corona radiata, and right bridge arm lesions (Figure 3). MRI of the whole spine revealed no lesion spinal cord (Figure 4) and MRI enhancement (Figure 5). Visual evoked potential (VEP) showed that the latency of each wave of VEP on the right side was longer than that of the opposite side. Brainstem auditory evoked potential (BAEP) revealed that the latency of V wave in the bilateral brainstem area was prolonged. Cerebrospinal fluid pressure of lumbar puncture was 140 mmH_2_O. Paneth's test (+), total protein, 1.06 g/L; immunoglobulin *G*, 100 mg/L; nucleated cell count, 25 × 10^6^/L; lymphocytes, 67%; and monocytes, 33%. Bacterial examination, acid-fast staining, and Chinese ink staining of the cerebrospinal fluid showed no abnormalities. There was no immunoglobulin *G* (IgG) in both the serum and cerebrospinal fluid. Antinuclear antibody spectrum, vasculitis spectrum, thyroid autoantibodies and related antibodies, antivasculitis antibody spectrum, anticardiolipin antibody (ACA), and other obvious abnormalities were not found. Tumor marker analysis showed carbohydrate antigen 72 − 4 11.8 U/mL and cytokeratin 4.06 ng/ml. Myelin sheath basic protein (MBP), 1.16 ng/mL; serum myelin sheath basic protein, MBP>1800 pg/mL; MBP Ab (+); and MOG Ab 1 : 320 (CBA) (+). Based on these findings, he was diagnosed as MOGAD and treated with oral methylprednisolone (1000 mg/d for 5 days), which was gradually decreased to 60 mg/d. Combined gamma globulin was administered at 0.4 g/(kg·d) for 5 days. The patient's symptoms improved significantly, he had clear consciousness, responded normally, fluent speech, and no apraxia. Neurological examination revealed good spirit, fluent speech, responsiveness, and memory and calculation power basically normal. The right limb muscle strength level was over level 4 and he was discharged from the hospital on February 11, 2018.

## 3. Discussion

At present, MOGAD is an independent disease different from MS and AQP4-IgG-positive NMOSD. The exact pathogenic mechanism of MOGAD is still unclear. Whether the antibody is pathogenic or only serves as a sign of myelin destruction is still inconclusive. As a myelin protein component present in the outermost layer of myelin in the CNS, MOG is involved in regulating the stability of oligodendrocyte microtubules and the integrity of the myelin structure and mediating the interaction between myelin and the immune system. Although MOGAD, MS, and AQP4-positive NMOSD overlap in clinical phenotype, the immunological and pathological manifestations are distinct. Acute NMO pathologically characterized as loss of AQP4, astrocytes, and oligodendrocytes. There was complement deposition and infiltration of eosinophils around vascular.

Previous reports have found that MOGAD is more common in children with ADEM-like manifestations, while in adults it is mostly ON [8]. The patient in this case had a subacute onset and worsened acute progression. Early manifestations were dizziness and weakness of the left limb. Further, MRI showed new focus in the right oval center and corona radiata. The onset type, symptoms, signs, and imaging findings of the patient are very similar to those of cerebral infarction. It is easy to misdiagnosed cerebral infarction. The patient's condition progressed and aggravated after treatments, new multiple bilateral lesions in both cerebral hemispheres indicating that the diagnosis was perhaps CNS demyelination, MBP in the serum and cerebrospinal fluid were elevated, and MOG antibody in serum was positive.

Since the clinical manifestations and imaging studies of MOGAD are not specific, diagnosis mainly relies on serum antibody detection, taking antibody detection early is helpful for diagnosis and treatment. The patient was diagnosed with MOGAD probably because of the positive detection of MOG antibody. Therefore, when we encounter such patients in clinical practice, we should broaden our clinical diagnostic criteria in a timely and effective manner to avoid missed diagnosis and misdiagnosis.

## 4. Conclusion

This case leads to that: when we encountered the patient's clinical manifestations and imaging findings resembling cerebral infarction, especially the treatments do not work well; we should broaden our thoughts and take antibody detection early avoiding misdiagnosed or missed diagnosis.

## Data Availability

The raw data supporting the conclusions of this article will be made available by the authors, without undue reservation.
